# Supplementary material for: Role of Clathrin Light Chains in Regulating Invadopodia Formation
Source: Cells. 2021 Feb 20;10(2):451. doi: 10.3390/cells10020451 (PMC7924216; doi:10.3390/cells10020451)
Supplement: Supplementary file 1 [file cells-10-00451-s001.zip › Suppl. Information/Suppl. Movies/Suppl movies.docx]

Suppl movie 1: Live CLCa back compensation. Live-cell confocal spinning disc microscopy of U373 CLCA-/- CLCB-/- CLCa GFP transfection 2 h before imaging (green) transiently expressing BacMam mcherry-lifeact (red). Live-cell confocal imaging was performed for 12h with a frame rate of 10 min/frame. The scale bar equals 20µm.

Suppl movie 2: U373 WT gelatin digestion. Live-cell confocal spinning disc microscopy of U373 WT cells stably expressing AP2 GFP (green) transiently expressing BacMam mcherry-zyxin (red) and seeded onto gelatin-647 (blue) coated coverslips. Live-cell confocal imaging was performed for 10h with a frame rate of 10 min/frame. The scale bar equals 20µm.

Suppl movie 3: U373 CLCA-/- CLCB-/- gelatin digestion. Live-cell confocal spinning disc microscopy of U373 CLCA -/- CLCB -/- cells stably expressing AP2 GFP (green) transiently expressing BacMam mcherry-zyxin (red) and seeded onto gelatin-647 (blue) coated coverslips. Live-cell confocal imaging was performed for 10h with a frame rate of 10 min/frame. The scale bar equals 20µm.
