# Supplementary figures and images for: Role of Clathrin Light Chains in Regulating Invadopodia Formation
Source: Cells. 2021 Feb 20;10(2):451. doi: 10.3390/cells10020451 (PMC7924216; doi:10.3390/cells10020451)

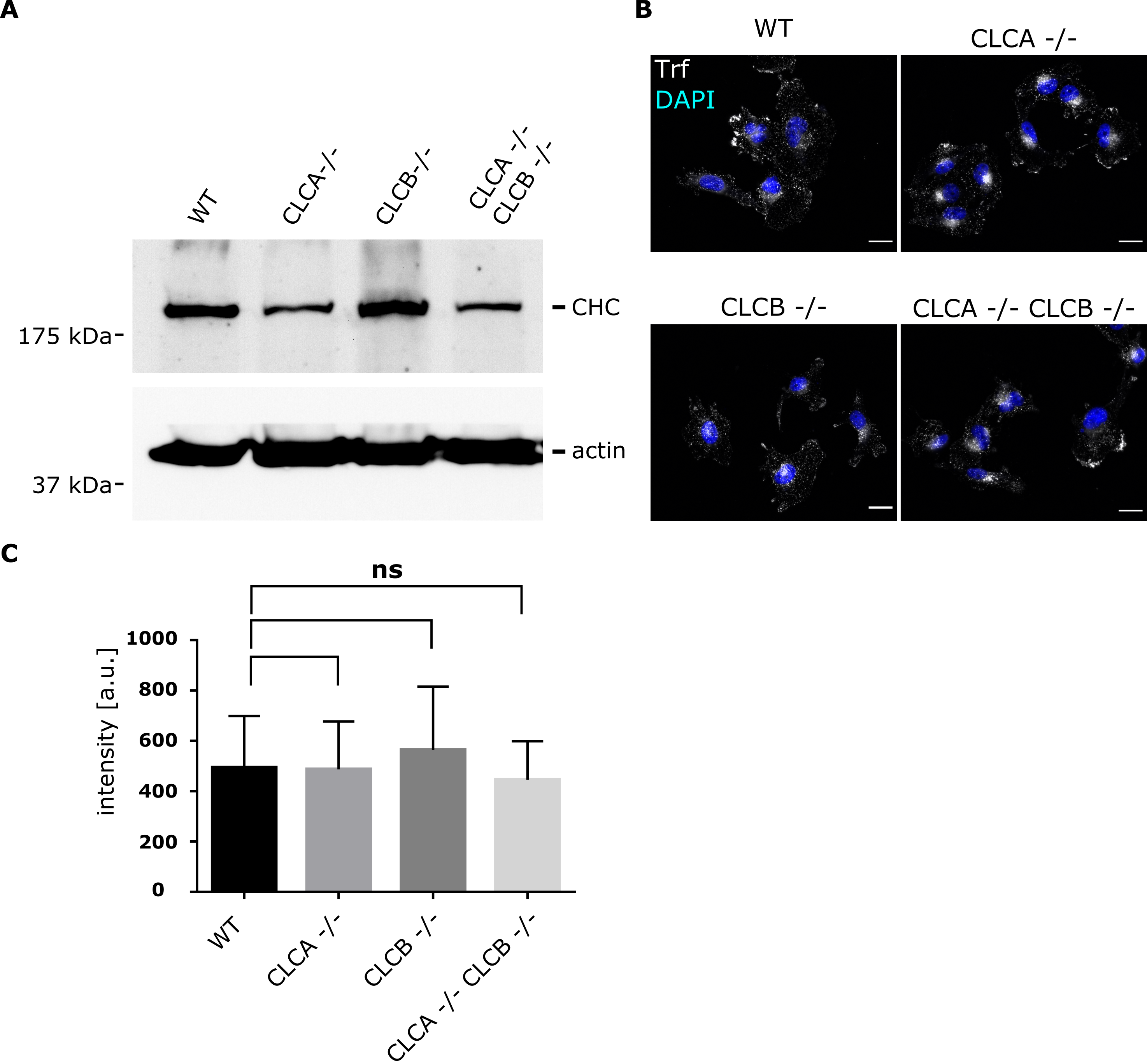

Supplement: Supplementary file 1 [file cells-10-00451-s001.zip › Suppl. Information/Suppl. Figure 1/Suppl. Figure 1.png]

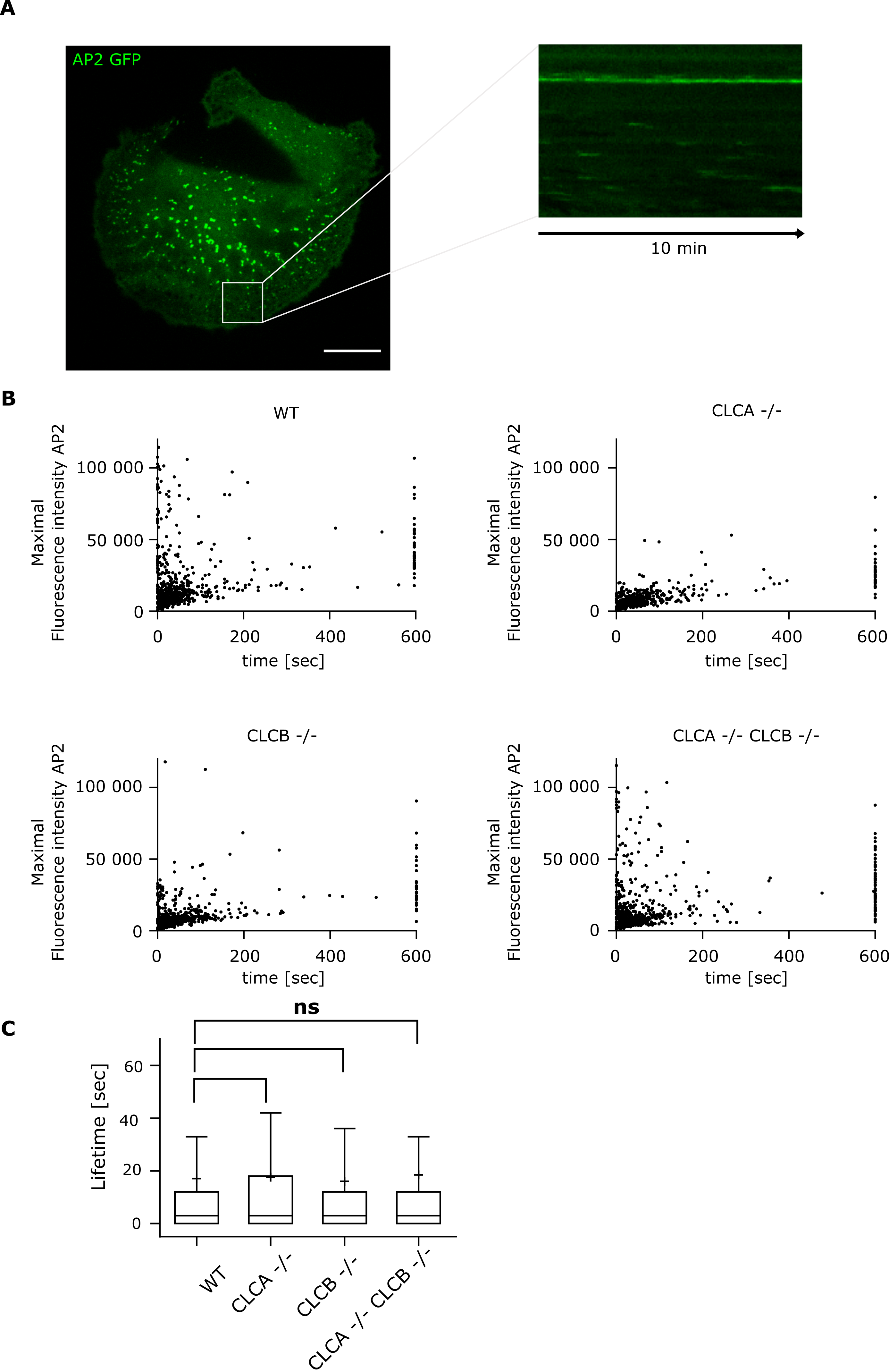

Supplement: Supplementary file 1 [file cells-10-00451-s001.zip › Suppl. Information/Suppl. Figure 2/Suppl. Figure 2.png]

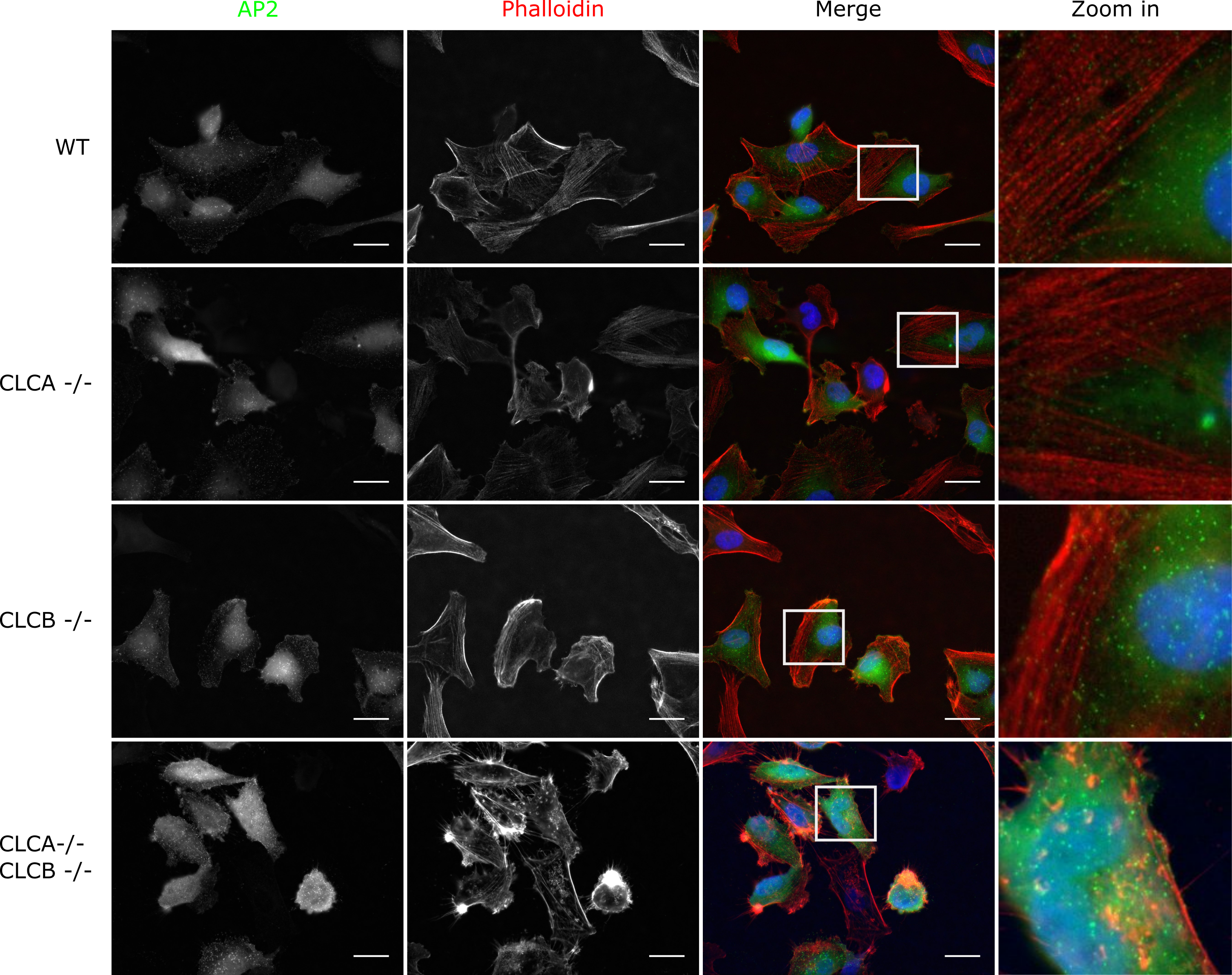

Supplement: Supplementary file 1 [file cells-10-00451-s001.zip › Suppl. Information/Suppl. Figure 3/Suppl. Figure 3.png]

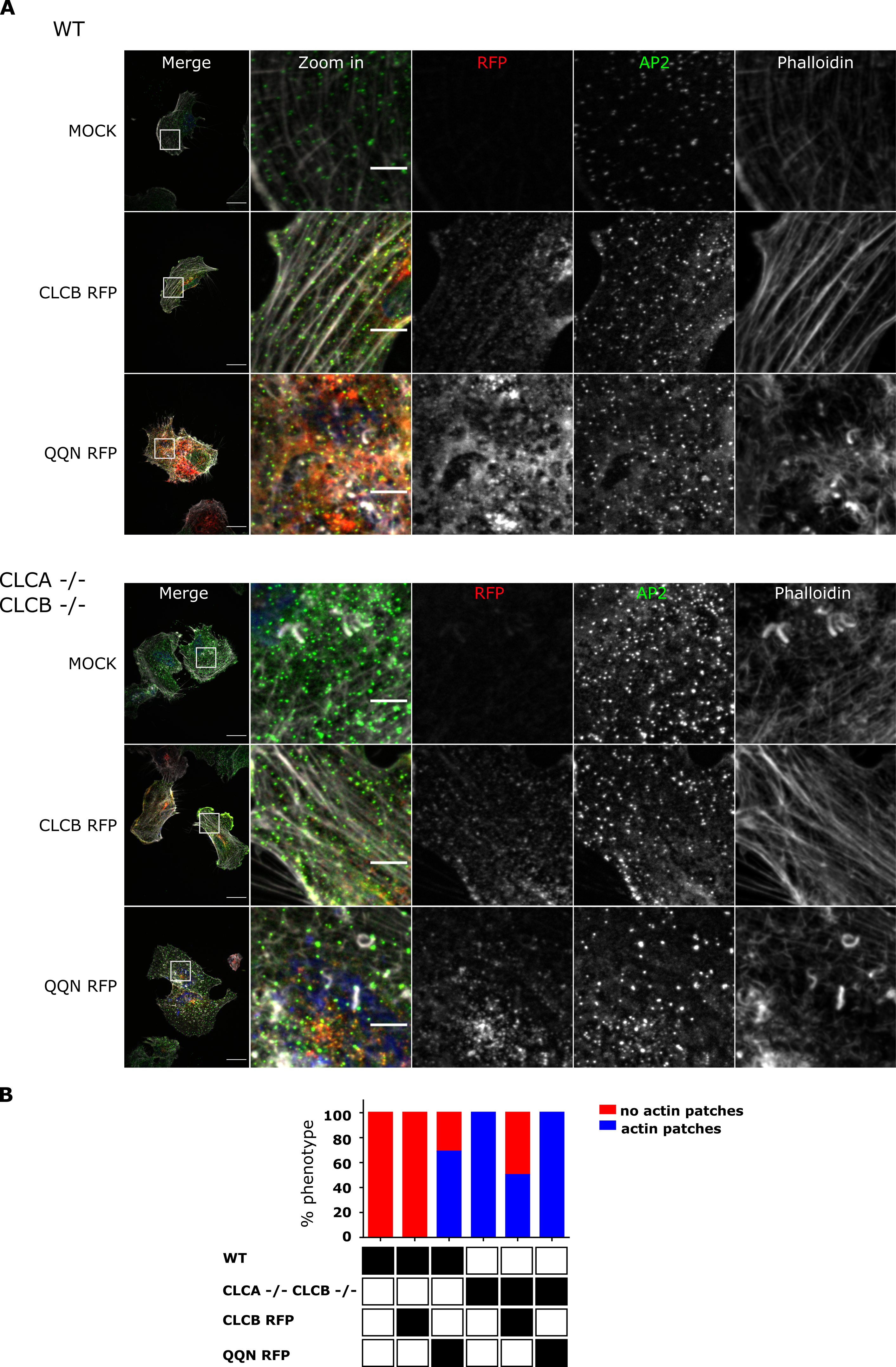

Supplement: Supplementary file 1 [file cells-10-00451-s001.zip › Suppl. Information/Suppl. Figure 4/Suppl. Figure 4.png]

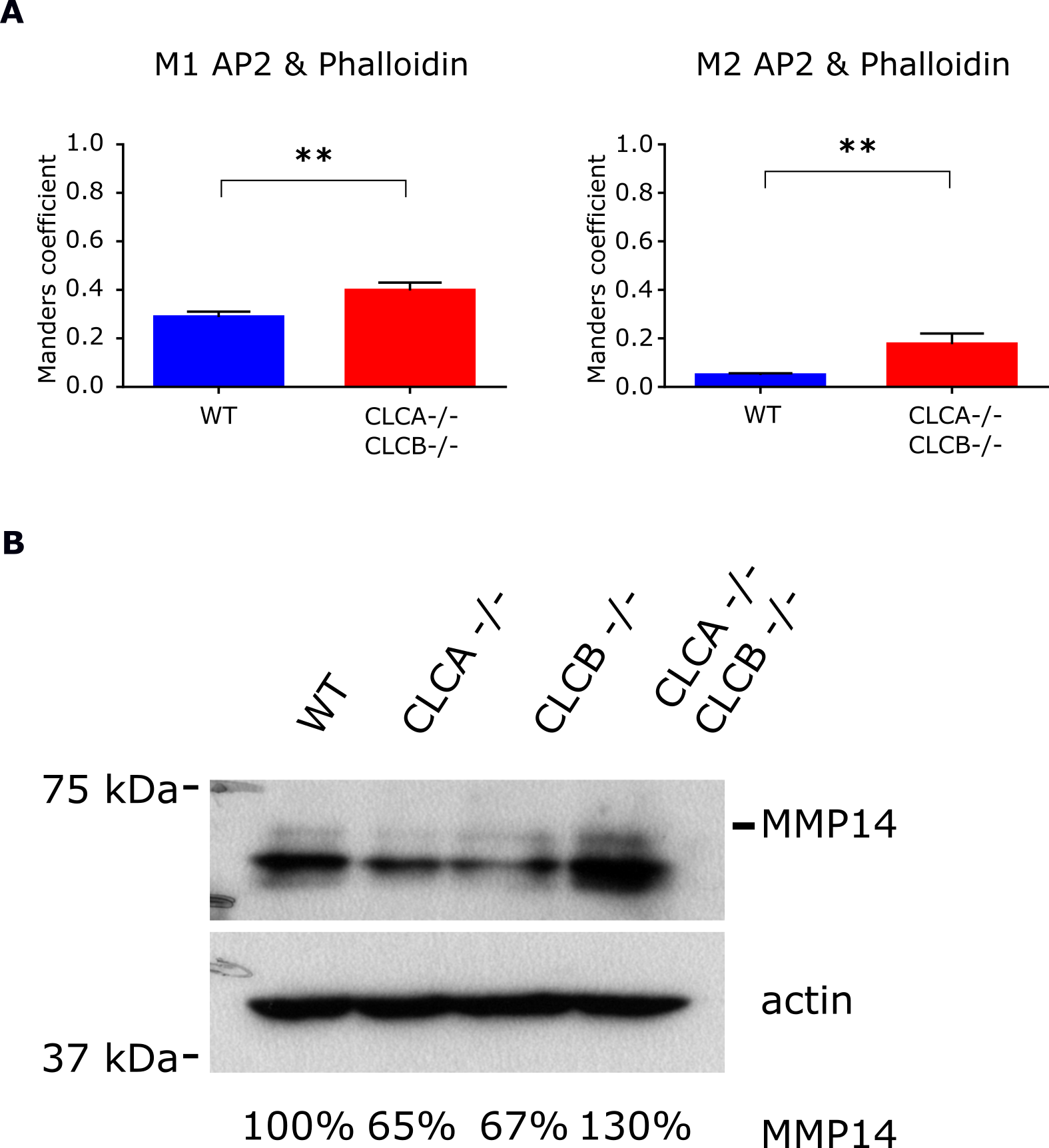

Supplement: Supplementary file 1 [file cells-10-00451-s001.zip › Suppl. Information/Suppl. Figure 5/Suppl.Fig 5.png]

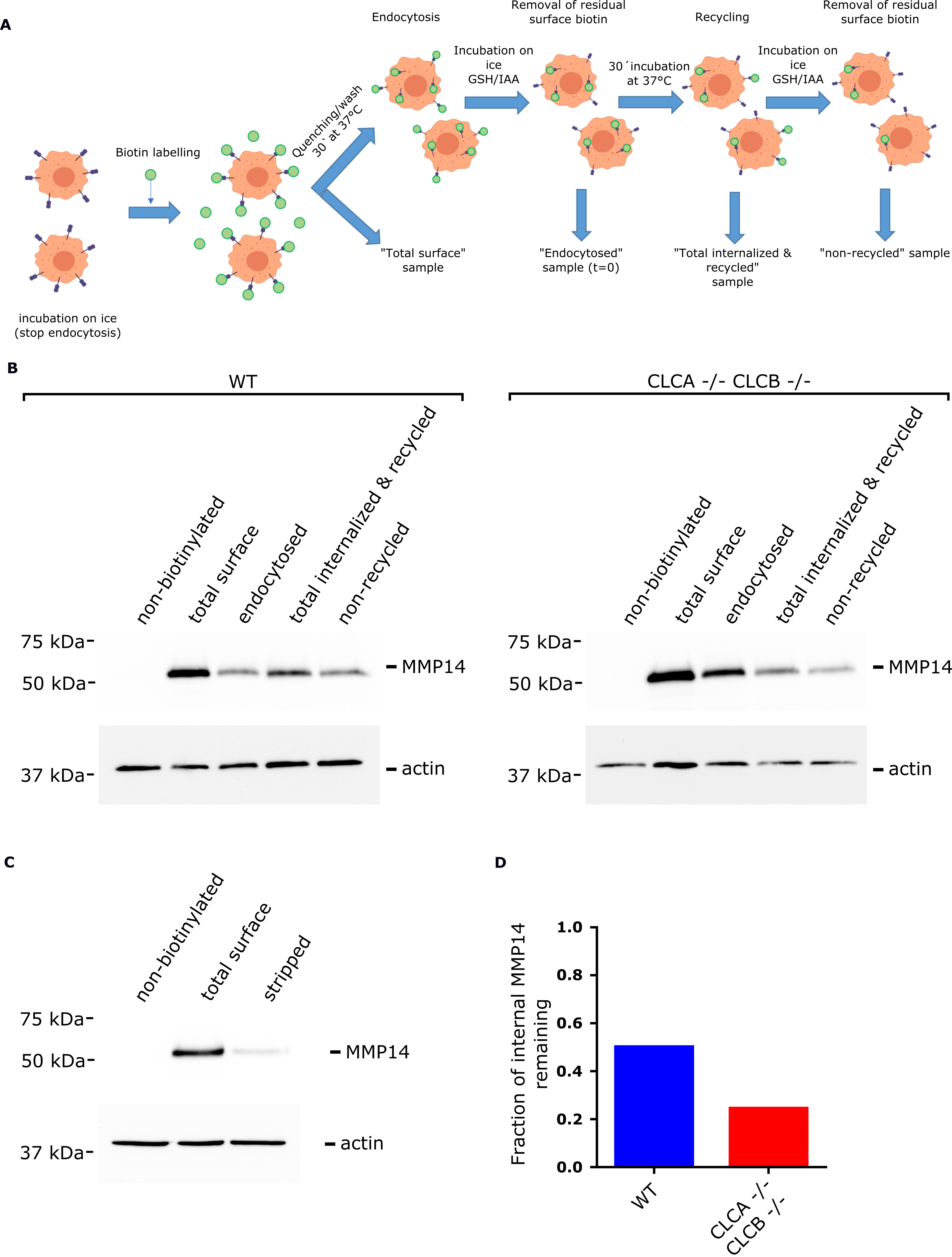

Supplement: Supplementary file 1 [file cells-10-00451-s001.zip › Suppl. Information/Suppl. Figure 6/Suppl. Figure 6.png]

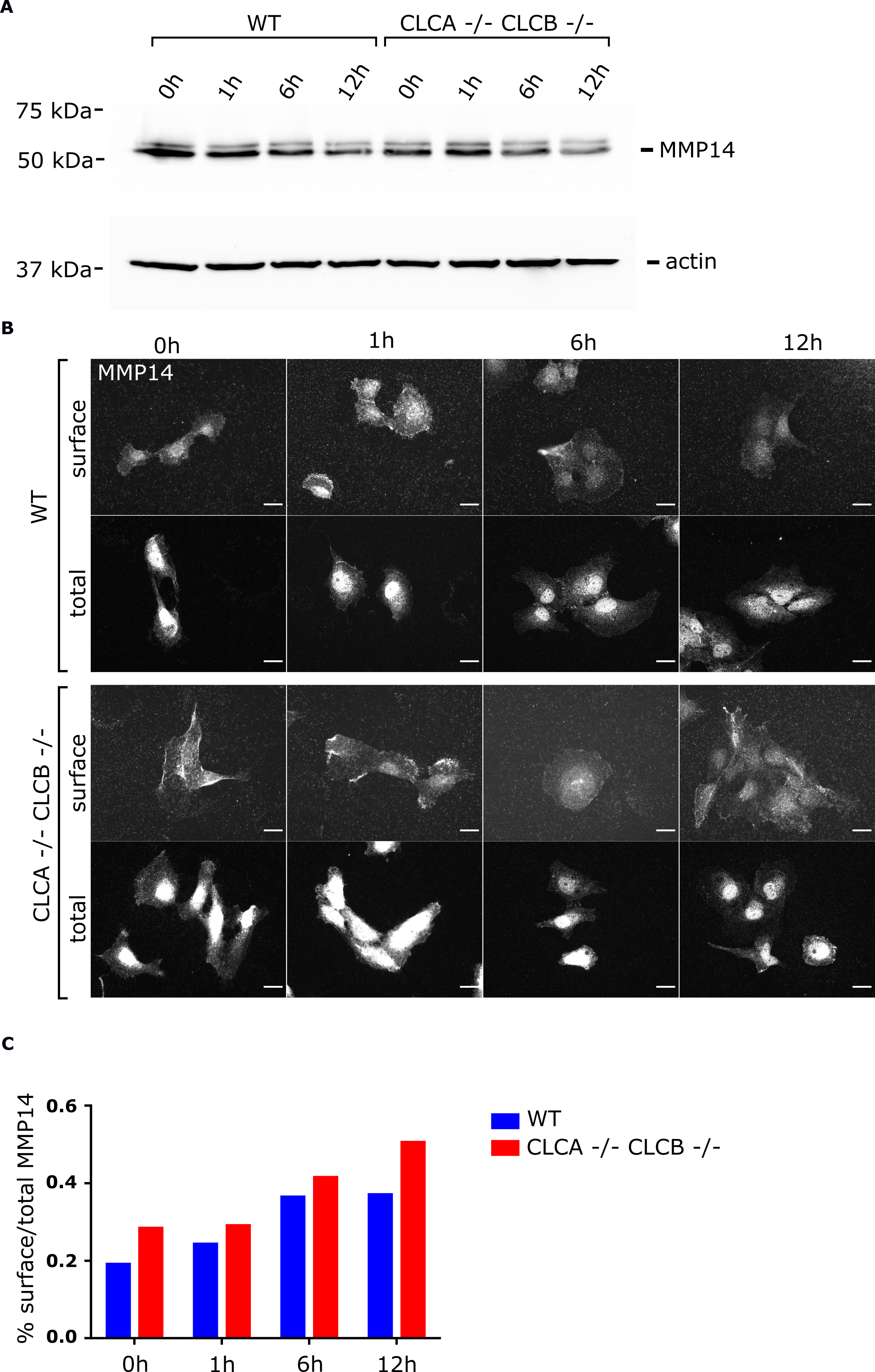

Supplement: Supplementary file 1 [file cells-10-00451-s001.zip › Suppl. Information/Suppl. Figure 7/Suppl. Figure 7.png]

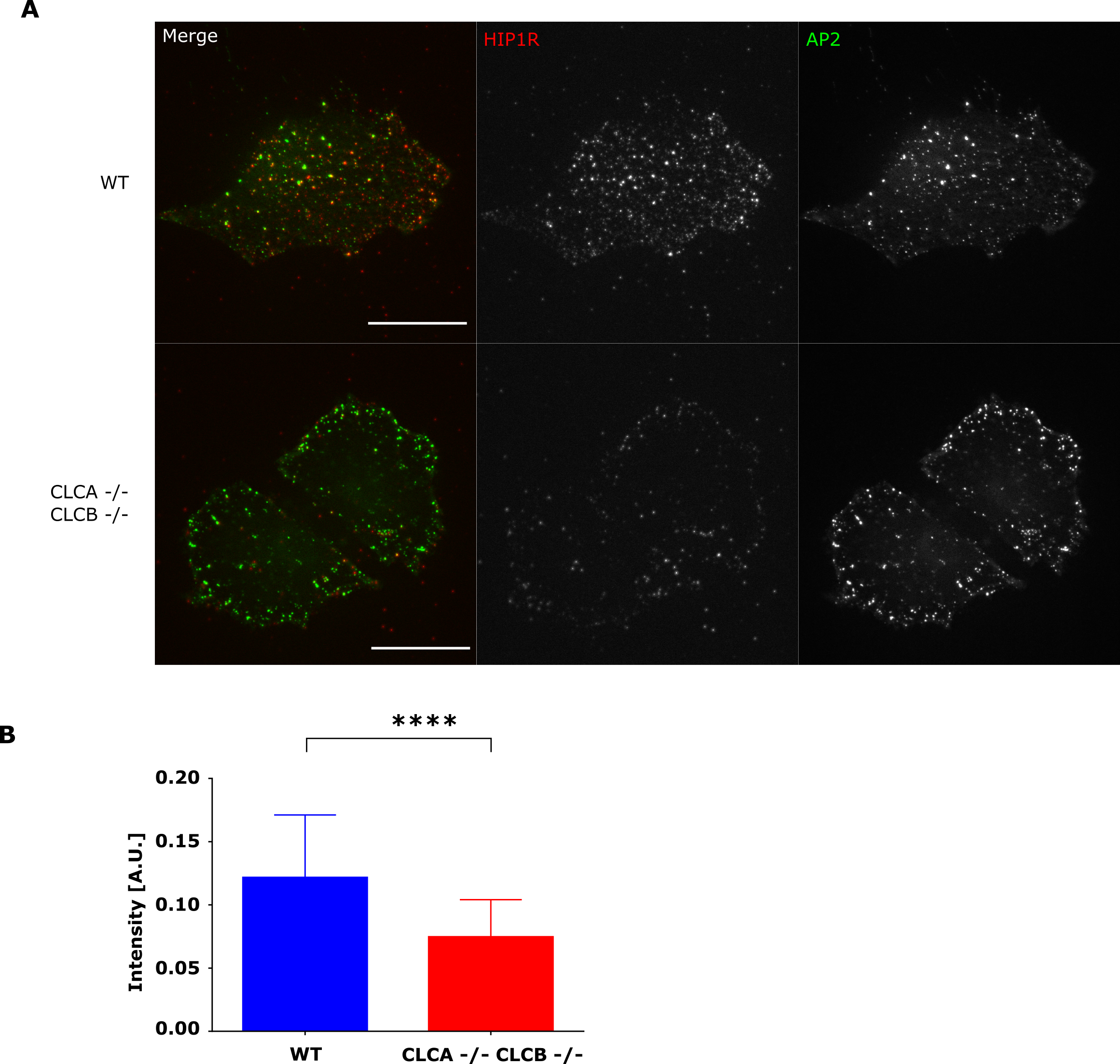

Supplement: Supplementary file 1 [file cells-10-00451-s001.zip › Suppl. Information/Suppl. Figure 8/Suppl. Figure 8.png]
